# Impact of clinical symptoms and diagnosis: the electronic Person-Specific Outcome Measure (ePSOM) development programme

**DOI:** 10.1186/s41687-022-00433-2

**Published:** 2022-04-05

**Authors:** S. Saunders, S. Sheehan, G. Muniz-Terrera, S. Luz, C. W. Ritchie

**Affiliations:** 1 Centre for Clinical Brain Sciences, University of Edinburgh, Edinburgh, UK; 2 Usher Institute of Population Health Sciences and Informatics; Molecular, Genetic and Population Health Sciences, University of Edinburgh, Edinburgh, UK; 3 Brain Health Scotland, Edinburgh, UK

**Keywords:** Clinically meaningful change, Electronic patient reported outcome measures, Alzheimer’s disease, Outcome measures, Brain health

## Abstract

**Introduction:**

Regulatory bodies recommend that outcome measures used in Alzheimer’s disease (AD) clinical trials capture clinically meaningful changes for the trial participant. However, commonly used outcome measures do not reflect the individual’s views on what matters to them individually. The aim of the electronic Person-Specific Outcome Measure (ePSOM) programme is to better understand what outcomes matter to patients in early Alzheimer’s disease.

**Methods:**

As part of the ePSOM programme, we designed and ran an online study to understand what matters to individuals when developing new treatments for AD. The ePSOM survey ran Aug 2019–Dec 2019 (UK) and collected primarily free text responses which were analysed using Natural Language Processing (NLP) techniques. In this paper, we focus our analyses on individuals who reported having a neurodegenerative disease diagnosis (primarily Mild Cognitive Impairment (MCI) or AD), reporting the most frequent and most important brain health priorities for this group. Due to a small sample size, the Diagnosis group was analysed as a whole. Finally, we compared the Diagnosis group to an age and gender matched control group using chi-squared tests to look for any differences between the Diagnosis and control groups’ priorities.

**Results:**

The survey was completed by 5808 respondents, of whom 167 (2.9%) (women n = 91, men n = 69, other n = 7) had received one of our pre-defined neurodegenerative disease diagnosis: most commonly MCI n = 52, 1.1% (mean age 69.42, SD = 10.8); or Alzheimer’s disease n = 48, 1.0% (mean age 71.24, SD = 9.79). Several thematic clusters were significantly more important for the target diagnostic group, e.g.: *Expressing opinions*; and less important, e.g., *Cognitive Games*.

**Conclusion:**

We conclude there are a range of outcomes which individuals consider important and what potential new treatments should help maintain or improve, suggesting that outcomes that matter shift along the preclinical, prodromal and overt dementia continuum. This has important implications for the development of outcome measures in long term prevention studies that last several years where participants may pass through different stages of disease. In the final stage of our project, we will design an electronic outcomes app which will employ the methodology tested in the large-scale survey to capture what matters to individuals about their brain health at an individual level.

**Supplementary Information:**

The online version contains supplementary material available at 10.1186/s41687-022-00433-2.

## Background

With a world-wide improvement in life expectancy, there is an increasing focus on maintaining brain health over the life course but particularly in old age in relation to age-associated diseases. This is a way of reducing the risk of developing neurodegenerative disorders such as Alzheimer’s disease (AD) as evidence suggests the underlying AD pathology starts years earlier than the dementia syndrome manifests [1, 2]. Therefore, the preclinical phase where AD pathology can be identified but when there is no clinical manifestation or the prodromal phase where any clinical decline is mild and does not impede daily living, offers an opportunity to prevent pathological changes from progressing further and ultimately dementia manifesting. Excluding clinical trials, there are currently no disease modifying treatments available for dementia though symptomatic relief may be effective for 1 or 2 years from the start of medication [3]. Accordingly, the emphasis on earlier disease identification and ultimately prevention of further decline, involves focusing on individuals who have early stages of brain disease but do not (yet) have dementia. Consequently, a move towards the earlier stage of the AD continuum necessitates being able to measure pathology, clinical change and meaningful outcomes in individuals who do not demonstrate dementia symptoms. However, while the scientific direction for AD research is to derive knowledge to facilitate interventions for an earlier disease stage, the majority of AD clinical trials still target the early symptomatic stage of the AD continuum where individuals have possible prodromal dementia (previously referred to as mild cognitive impairment) or early dementia. Easier recruitment and novel outcome measures need to be developed to encourage the undertaking of clinical trials in this earlier population. The ePSOM programme seeks to develop an outcome measure that is considered clinically meaningful and has greatest utility in the pre-dementia stages of neurodegenerative disease. A key methodology of our programme was to use Natural Language Processing to allow analysis of a vast amount of free text data on what matters to individuals about their brain health [4]. This is the route that will allow unrestricted expression of treatment priorities by the individual using free written text (or speech) to define the outcome as opposed to a restricted choice which is characteristic of traditional functional and Quality of Life measures often used as proxies of ‘clinically meaningful’. While our initial analyses provided evidence that individuals’ priorities may vary significantly based on demographic differences [4], we now explore whether priorities may also vary based on where individuals are on the neurodegenerative disease spectrum.

Even though the overall cost in AD drug development is remarkably higher than other disease areas [10], almost all trials over the last 2 decades have resulted in failures [5]. Aducanumab was the first AD drug to receive FDA approval in the last 2 decades [6] with the primary outcome a reduction of amyloid. One of the criticisms of failed drug studies is focusing on individuals whose pathological changes are too advanced [5, 7, 8], but equally employing outcome measures designed for a later disease stage which may not be sensitive at such an early disease stage [9, 10]. Historically, AD clinical trials centre around measuring change in cognition [11] but arguably, the disease stage where cognition is affected to the extent where it can be measured using traditional cognitive assessments, is further down the disease spectrum and also at a point where any interventions are less likely to be effective due to the advanced disease stage. It is critical to innovate AD trial designs which means identifying the appropriate preclinical or prodromal study participants and employing relevant outcome measures [7]. There are a number of drugs in development [12] currently and noticeably, research increasingly focuses on the pre-dementia stages of the disease continuum with an on-going six phase 3 trials targeting the earliest preclinical population although the primary outcome measures still focus on cognition (with secondary measures focusing on biomarkers or function) [13].

Guidance from the US Food and Drug Administration (FDA) as well as the European Medicines Agency (EMA) recommends using outcome measures in the pre-dementia stages which capture meaningful change for the individual [14, 15]. Evidence from studies looking into what matters to individuals with early AD or dementia [16–18] suggests there is heterogeneity in what individuals consider important and our previous work has shown there are items, such as confidence [19] which are currently not captured in outcome measures. Depending on the target population of the clinical trial, there is a need to incorporate the views of individuals along the AD continuum, so any benefit of a drug is corroborated by a meaningful effect noticed by the individual taking the medication.

Patient reported outcome measures (PROMs) are recognised as offering the necessary insight and adding the individual’s own perception of well-being. As part of the electronic Person Reported Outcome Measure (ePSOM) development programme [20], we aim to create an electronic version of such measure which would be specific and sensitive to the individual using it when assessing their own brain health and treatment priorities. Despite the regulators’ call for including PROMs in clinical trials for AD, no measures have been used consistently in regulatory studies. Functional measures focus on skills typically needed to manage basic physical needs [21] such as those assessing Activities of Daily Living (ADLs) or Quality of Life, may not always be applicable to individual participants and they are subject to cultural or societal expectations in deriving a score (e.g., all individuals may not have the same skills to manage their physical needs regardless of disease presence). However, PROMs offer an opportunity to report change which is personally meaningful and relevant for the individual. We developed the ePSOM programme to obtain evidence around what outcomes matter to individuals about their brain health with an ultimate aim to create a personalised outcome tool. An electronic platform would allow individualising the outcome measure to capture what matters to the person and used in parallel with biological measures of AD, an ePSOM could provide a secondary endpoint to offer further proof of drug effectiveness from the study participant’s point of view. In this paper, we specifically analyse the answers from individuals at a more advanced stage of the neurodegenerative disease spectrum in order to reflect whether these priorities of brain health are currently captured in AD clinical trials.

## Methods

We designed and ran a UK-wide population-based online study collecting primarily free text answers. The methods, the survey itself as well as the overall results are described in more detail elsewhere [4]. In brief, the ePSOM study ran from Aug 2019 to Dec 2019 and was divided into sections where respondents were asked to provide free text answers on what they would like to retain or keep being able to do if their brain health got worse. At the end of the study, respondents were asked to identify no more than five answers across all the answers they had given which they consider the most important. The study obtained ethics approval from the ACCORD Medical Research Ethics Committee in Edinburgh, Scotland.

The survey was open to anybody over the age of 18 and advertised primarily via Alzheimer’s Research UK communication channels. While the study employed primarily qualitative free text methods, we also collected key sociodemographic and clinical data such as self-reported diagnosis related to brain health and taking anti-dementia medication. We used Natural Language Processing (NLP) techniques to analyse the free text data. We considered it critical that respondents were not limited in predefined answers but had the ability to freely express anything that is relevant for them personally about their brain health within five domains [1], Everyday functioning [2]; Sense of Identity [3]; Relationships and Social Connections [4]; Enjoyable Activities and [5] Thinking problems.

### Natural language processing

To reduce the large diversity of distinct free-text answers collected in survey (n = 82,514 answers; 460,906 words) to a manageable number of themes we employed word embedding and clustering methods. Semantic representations (vectors) for the answers were generated using GloVe embeddings pre-trained on a corpus of 6 billion words [22]. These vectors were used to cluster semantically similar text segments. This allowed us to use automated methods to identify words, and thus answers, of a similar “theme” or meaning. The K-means clustering algorithm was used to cluster the answer embeddings within each of the five domains. The K parameter, that is, the desired number of automatic clusters per domain, was determined analytically using the elbow method. This method resulted in a total of 755 clusters of free text answers, or 151 clusters for each of the five domains.

These clusters were reordered within each of the five domains so that semantically similar clusters appeared close together. This was achieved using hierarchical clustering on the cluster centroids. We used the reordered clusters for manual annotation in each of the five domains. Six authors of the current paper annotated 2 domains each, ensuring two separate people analysed a single domain as well as assess inter-rater reliability between domains. Finally, two of the authors did quality control across the five domains and homogenised the labels across domains.

### Statistical analyses

In this paper, we focus our analyses on individuals who reported having a brain health related diagnosis. Specifically, these are respondents who self-reported having one or a combination of the following diagnoses in our pre-defined neurodegenerative disease list: Mild Cognitive Impairment; Alzheimer’s disease; Vascular dementia; Frontotemporal dementia; Mixed dementia; Parkinson’s disease; Dementia with Lewy bodies; Korsakoff syndrome; Motor Neurone Disease. Six respondents reported having a diagnosis, but they were not sure what their diagnosis was. However, as they also reported taking anti-dementia medication, we included those six respondents in the Diagnosis group.

We analysed data from this group of respondents as one Diagnosis group rather than split the group by different diagnoses. This was done because the individual diagnosis groups would be small relative to the entire study population and we wanted to avoid multiple comparisons and Type I statistical error. We also considered the limitations of self-reported diagnoses as this is vulnerable to inaccurate reporting. Finally, as the majority in the diagnosis group were individuals with MCI or AD, the distinction between mild impairment and early AD is not clear in the absence of standardised diagnostic criteria.

First, we identified all respondents in the Diagnosis group and used a t-test to determine if the group’s sociodemographic characteristics are significantly different from the rest of the study sample. We then controlled for age (as the Diagnosis group was significantly older than the rest of the survey sample) and gender (as the Diagnosis group had significantly more men) and created a reference group from the rest of the survey sample to ensure that any differences in ‘what matters to individuals about their brain health’ would not be driven by age or gender (referred to as control group in the paper). We used propensity score matching without repetition to create the matched sample (control group), meaning there is no statistical difference in age and gender proportions between the Diagnosis and the control groups.

We present the most important outcomes that matter to individuals in ‘themes’ with [1] the largest themes, representing answers which were given the most frequently; and [2] themes which were identified as *particularly important,* representing answers from the end of the survey where respondents were forced to pick no more than five top important outcomes across all the answers they had given.

Finally, we also conducted Chi-squared tests to evaluate whether the differences in percentages between the Diagnosis group’s answers within each theme were statistically significantly different from respondents in the control group. A *p*-value of < 0.01 was used in statistical significance testing.

## Results

### Description of groups as per key characteristics

A total of 5808 respondents took part in the study. We identified 167 individuals (2.88%) who self-reported a neurodegenerative disease diagnosis (the Diagnosis group). The total number of free text answers given by the Diagnosis group was 1976 (2.39% of all the survey answers). The descriptive statistics of the diagnosis group is presented in Table 1 including significant differences between the Diagnosis and control group.

**Table 1:** Descriptive statistics of the diagnosis group and the age and gender matched reference group

	Diagnosis group		Control group	
Number of respondents	n = 167		n = 1002 Controlled	
*Men*	*n* = *83*		*n* = *462 Controlled*	
*Women*	*n* = *84*		*n* = *540 Controlled*	
Diagnosis	MCI n = 52		No diagnosis Controlled	
	Alzheimer’s disease n = 48			
	Vascular dementia n = 19			
	Combination^†^ n = 42			
	Not sure but taking anti-dementia medication n = 6			
Age	Mean: 70.2 (SD = 10.04)		Mean: 69.8 (SD = 9.84) Controlled	
Education levels	Degree or higher	n = 83	Degree or higher	n = 614*
	No degree	n = 84	No degree	n = 388
Living area	Urban	n = 102	Urban	n = 614
	Rural	n = 63	Rural	n = 377
	Prefer not to say	n = 2	Prefer not to say	n = 11
Did anyone assist you with filling out the survey?	Yes	n = 53*	Yes	n = 7
	No	n = 114	No	n = 995
Taking anti-dementia medication	Yes	n = 72*	Yes	n = 0
	No	n = 95	No	n = 1002

Values which are statistically more likely are marked with *

^†^MCI and Other (n = 7); Frontotemporal dementia (n = 7); Mixed dementia (n = 4); Alzheimer’s disease and Vascular Dementia (n = 3); MCI and Alzheimer’s disease (n = 2); Subjective cognitive disorder (n = 2); Parkinson’s disease (n = 2); Dementia with Lewy bodies (n = 2); Alzheimer's disease, Mixed dementia, Vascular dementia (n = 1); MCI and Mixed dementia (n = 1); Alzheimer's disease, Mixed dementia (n = 1); Mixed dementia, Vascular dementia (n = 1); MCI and Vascular dementia (n = 1); Alzheimer's disease, Vascular dementia, Not sure (n = 1); Mixed dementia, Frontotemporal dementia (n = 1); Frontotemporal dementia, Korsakoff syndrome (n = 1); Parkinson's disease dementia (n = 1); Parkinson's disease dementia, Parkinson's disease (n = 1); Korsakoff syndrome (n = 1); Vascular dementia, Parkinson's disease (n = 1); MCI, Alzheimer's disease, Mixed dementia, Vascular dementia, Not sure (n = 1)

### The most frequent and most important themes for the diagnosis group

The NLP analysis resulted in 150 themes of importance about brain health (*see * Table 2). While the most popular theme for both frequent and important themes is *Driving*, there are differences in what is mentioned often versus what is considered top priority (as respondents were asked to select no more than five of their previously given answers as most important).

**Table 2:** This table shows the top 35 themes which were mentioned the most and the top 35 themes which were picked as particularly important by the Diagnosis group

	Theme (by frequency)	Count (%)	Theme (by importance)	Count (%)
1	Driving	102 (5.16%)	Driving	60 (8.00%)
2	Socialising	65 (3.29%)	Family Connection	30 (4.00%)
3	Reading	64 (3.24%)	Conversation and Chat	26 (3.47%)
4	Walking	62 (3.14%)	Reading	24 (3.20%)
5	Follow a storyline	60 (3.04%)	Friendships	24 (3.20%)
6	Friendships	59 (2.99%)	Walking	24 (3.20%)
7	Family connection	51 (2.58%)	Socialising	22 (2.93%)
8	Conversation and chat	48 (2.43%)	Mix Family Connections, Friendships	17 (2.27%)
9	Gardening	43 (2.18%)	Remembering	15 (2.00%)
10	Cooking	35 (1.77%)	Maintain Independence	15 (2.00%)
11	Mix Family Connections, Friendships	34 (1.72%)	Communicate Effectively	13 (1.73%)
12	Use Technology	30 (1.52%)	Use Technology	12 (1.60%)
13	Remembering	27 (1.37%)	Feel Wanted and Needed	12 (1.60%)
14	Remember Past	26 (1.32%)	Gardening	10 (1.33%)
15	Recognise People	26 (1.32%)	Make Decisions	10 (1.33%)
16	Feel Wanted and Needed	26 (1.32%)	Cooking	10 (1.33%)
17	Shopping	26 (1.32%)	Dining	9 (1.20%)
18	Understand current affairs	23 (1.16%)	Remember Past	9 (1.20%)
19	Communicate Effectively	21 (1.06%)	Exercise	9 (1.20%)
20	Give Advice	20 (1.01%)	Maintain Dignity	8 (1.07%)
21	Personal Attributes and Social Skills	20 (1.01%)	Singing	8 (1.07%)
22	Hobbies	19 (0.96%)	Follow a storyline	8 (1.07%)
23	Maintain Independence	19 (0.96%)	Hobbies	8 (1.07%)
24	Analyse and Solve Problems	19 (0.96%)	Meaningful Conversations	8 (1.07%)
25	Singing	18 (0.91%)	Recognise People	7 (0.93%)
26	Confidence	18 (0.91%)	Grandchildren	7 (0.93%)
27	Meaningful conversation	18 (0.91%)	Shopping	7 (0.93%)
28	Grandchildren	18 (0.91%)	Golf	6 (0.80%)
29	Dining	18 (0.91%)	Go on Holidays	6 (0.80%)
30	Music	17 (0.86%)	Volunteering	6 (0.80%)
31	Express Opinions	16 (0.81%)	Walk dogs	6 (0.80%)
32	Manage Finances	16 (0.81%)	Cognitive Games	6 (0.80%)
33	Make Decisions	16 (0.81%)	Live at home	5 (0.67%)
34	Help others	16 (0.81%)	Mix Board Games and Cards	5 (0.67%)
35	Exercise	16 (0.81%)	Pets	5 (0.67%)
	*Total answers in group*	*1976 (100%)*	*Total answers in group*	*750 (100%)*

Full figure of themes with the most Frequent and most Important answers in Additional file 1: Appendix 1, Additional file 2: Appendix 2

### Significant differences between themes. Diagnosis group versus control group

The Chi-squared tests to evaluate whether the differences in percentages between the Diagnosis group’s answers within each theme were statistically significantly different from the control group (matched for age and gender) showed there were six themes in the most frequently mentioned themes and four theme in the most important themes which were significantly different between the two groups (see Table 3).

**Table 3:** Themes which were mentioned the most which had the highest Chi square values representing greater differentiation between the Diagnosis group and control group AND Themes selected as particularly important which had the highest Chi square values representing greater differentiation between the Diagnosis and control group

	Theme (frequency)		*P*-value (< 0.01)	Chi square	Theme (importance)		*P*-value (< 0.01)	Chi square
1	Express Opinions	↑	< 0.01	14.69	Organise Home	↑	< 0.01	13.27
2	Cognitive Games	↓	< 0.01	13.55	Manage Finances	↓	< 0.01	7.72
3	Remembering	↑	< 0.01	13.19	Driving	↑	< 0.01	6.53
4	Driving	↑	< 0.01	6.85	Conversation and Chat	↑	< 0.01	6.16
5	Travelling	↓	< 0.01	6.77				
6	Manage Finances	↓	< 0.01	6.77				
	*Total answers in group*		*183*		*Total answers in group*		*101*	

↑Representing themes where respondents in the Diagnosis group are more likely to either report this theme as something that matters to them (Frequency) or select this theme as one of the most important themes (Importance)

↓Representing themes where respondents in the Diagnosis group are less likely to either report this theme as something that matters to them (Frequency) or select this theme as one of the most important themes (Importance)

## Discussion

The move towards detecting underlying AD pathology at an earlier disease stage necessitates measuring change in clinical trials on a much more outwardly healthy study population. With this shift in clinical trials, it is important to employ outcome measures both sensitive to change but also capturing meaningful change for the individuals receiving an interventional treatment. While the overall aim of the ePSOM study was exploring what matters most to people about their brain health [4], here we focus our analyses on individuals with a neurodegenerative disease diagnosis. The study results showed significant differences in prioritises for people with and without a diagnosis in what they consider important about their brain health. Characterising the individuals in our study sample, we found that while controlling for age and gender, the group with a neurodegenerative disease diagnosis was significantly more likely to have lower educational attainment. Although this is in line with systematic reviews looking at education and risk of dementia where lower education has been found to be associated with a greater risk for dementia [23], this effect in our study may have been due to sampling (recruitment method of online-survey primarily reaching individuals with higher education levels).

Interestingly, we found a number of themes which the diagnosis group was significantly more likely than the control group to consider important about their brain health which are not captured by commonly used outcome measures in AD clinical trials—such as *Expressing Opinions*. Agency is an important consideration along the whole AD continuum [24] and promoting a sense of agency may improve well-being in older adulthood [25]. However, we note there were also themes which are commonly captured by ADL measures (such as *Managing Finances)* but which people with a neurodegenerative disease diagnosis were less likely than those without a diagnosis to consider important*.* Being less likely to report *Managing Finances* and *Traveling* as an important outcome may again link with agency or suggest a perceived loss of agency. On the other hand, *Cognitive Games* may represent an activity which individuals have either given up on or do not see the value in even though this is a preventative strategy for protecting brain health.

Comparing the results from our study to gold standard assessments in AD trials reveal there are many items which are currently not captured by the traditional measures developed for staging dementia in a more clinically advanced population. Crucially, often assessments rely on the study partner’s perspective, but this is not consistent with the scientific understanding of how the preclinical or prodromal phase of AD outwardly presents. A commonly used assessment, and one which showed improvement in the recent approval of the Aducanumab drug [6], measuring global functioning in AD trials is the Clinical Dementia Rating Scale (CDR) [26]. The CDR is scored first by interviewing the study partner, followed by the study participant in order to assess accuracy of the study participant’s account of the same information previously provided by the study partner. The results from our study show that only a few of the top 35 themes which matter to people with a diagnosis are included in the CDR and even then, the major contribution towards the overall score of CDR is derived from the point of view of the study partner rather than the individual’s own perception of what matters to them. Crucially, the CDR is focused on staging dementia from [1] no impairment to [2] MCI, followed by more advanced stages of dementia which makes is unsuitable as a sensitive measure for the pre-dementia stages of illness. Our results provide evidence that there are important internal processes which matters to individuals with a diagnosis which may not be detected from either an outsider’s perspective or when focusing on functional assessments such as ADLs alone. Accordingly, *Feeling Wanted and Needed* and *Communicating Effectively* are in line with our earlier work [19] where we identified *Sense of Identify* as one of the main themes which emerged as something that matters to individuals (for individuals both with and without memory concerns or a diagnosis).

Even though the AD trials are moving to an earlier disease stage, there is still a suggestion to involve a study partner even in the case of pre-dementia studies [27]. This is despite evidence that accuracy and amount of information provided by study partners may vary [28, 29] and the study partner’s executive skills may influence the accuracy of subjectively reported ADLs [30] as well as perceived burden on reporting in global assessment scales [31]. Incorporating the individual’s own view of well-being is important as there are suggestions individuals in the asymptomatic pre-dementia stages of the AD continuum do experience decline from their own baseline and the combination of biomarker assessments with subjective assessments of subtle change may be useful in this group [32]. Fundamentally, an intervention’s success should reflect the abilities or skills that matter to the individuals and which they want to maintain. A need to incorporate an individual’s own perception of well-being alongside biological markers is evidenced by our study results showing the top themes emerging from our study may not be well captured in commonly used outcome measures; also, there are a range of views in what outcomes matter to individuals about their brain, illustrating the need for person specific outcome measures.

While the data in this study were collected relying on self-reported diagnosis, in the future when it is possible to use the ePSOM tool on better defined populations such as trial participants with a biomarker confirmed AD, we will be able to map out change measured by the ePSOM app which may correspond with biomarker change [20]. An ePSOM app could be administered in clinical trials once the individual’s eligibility is confirmed, before study randomisation and then through the course of the trial. Importantly, the ePSOM tool also does not limit itself to a stage of disease or cultural background. Nevertheless, we recognise the psychometric challenges involved with ensuring such an outcome measure is robust and comparable both in-person and on a group level.

The overall limitation of our study was the online study design that could have resulted in potential biases in responses due to self-selection. However, we note that the results of the study would not form the basis of an outcomes app but rather give evidence of the heterogeneity of priorities about brain health. The online design allowed us to develop NLP methodologies for analysing large-scale free text data which in turn will inform the technical aspects of the ePSOM app. Another limitation of our study is relying on self-reported diagnosis which in the absence of standardised clinical assessments may not be accurate. We mitigated against this by analysing the whole sample together who reported one of our target diagnoses rather than split these groups per diagnosis. However, the self-report nature of the study means the comparison group of people who did not report a neurodegenerative diagnosis may still contain an unknown number of respondents who are cognitively impaired but undiagnosed. The strength of our study was a well set-up programme of experiments using a step-by-step methodology with each previous stage of the programme informing the next. We used a novel AI based analytical approach, and finally we believe that the results show respondents understood the concept of the questions (*maintenance* of brain health) implying face validity of the outcome measure.

## Conclusions

In the ePSOM development programme, we have taken a systematic and evidence-based approach to understanding what matters to people about their brain health and how this could influence the outcomes used in AD clinical trials in the future when the target population is an at-risk group of individuals. Based on a literature review and a focus group study, we undertook a large-scale online survey employing novel NLP techniques enabling free text data analysis. Our approach allowed respondents to freely express priorities about their brain health and we have derived results based on the whole survey of nearly 6000 respondents [4] as well as in this paper focusing on the population with a clinical brain health diagnosis. The population of our study was mixed in terms of neurodegenerative disease diagnoses and while AD is the most common cause for dementia, the ePSOM tool could be employed to track meaningful outcomes in other neurodegenerative conditions too. As we understand more about the earliest stages of disease and put in place clinical services to identify and manage people at these early stages, clinical trials must use outcome measures that are ecologically valid, sensitive and specific to the stage of disease. Employing measures which capture personally meaningful change should not deter trials from enrolling patients very early in the course of disease. Biological measures should be used as primary outcomes at the early stages of the disease where underlying pathology has not yet become symptomatic but an intervention may delay or prevent symptoms from ever occurring. Alongside biological markers, robust, reliable and valid person specific outcomes tools could be used to measure the intervention’s effectiveness. The next phase of the ePSOM programme is to develop such a tool.

## Supplementary Information

**Additional file 1: Appendix 1.** Full list of most Frequent themes (Diagnosis group).

**Additional file 2: Appendix 2.** Full list of most Important themes (Diagnosis group).

## Data Availability

The datasets generated and analysed during the current study are not publicly available as free-text data may be difficult to entirely de-identify for public sharing but are available from the corresponding author on reasonable request.

## References

[1] Beason-Held LL, Goh JO, An Y, Kraut MA, O'Brien RJ, Ferrucci L (2013). Changes in brain function occur years before the onset of cognitive impairment. J Neurosci: Off J Soc Neurosci.

[2] Korte N, Nortley R, Attwell D (2020). Cerebral blood flow decrease as an early pathological mechanism in Alzheimer's disease. Acta Neuropathol.

[3] Casey DA, Antimisiaris D, O'Brien J (2010). Drugs for Alzheimer's disease: are they effective?. P & T: Peer-Rev J Formul Manag.

[4] Saunders S, Muniz-Terrera G, Sheehan S, Ritchie CW, Luz S (2021). A UK-wide study employing natural language processing to determine what matters to people about brain health to improve drug development: the electronic Person-Specific Outcome Measure (ePSOM) Programme. J Prev Alzheimer's Dis.

[5] Mehta D, Jackson R, Paul G, Shi J, Sabbagh M (2017). Why do trials for Alzheimer's disease drugs keep failing? A discontinued drug perspective for 2010–2015. Expert Opin Investig Drugs.

[6] Cummings J, Aisen P, Lemere C, Atri A, Sabbagh M, Salloway S (2021). Aducanumab produced a clinically meaningful benefit in association with amyloid lowering. Alzheimer's Res Ther.

[7] Siva N (2021). New global initiative to tackle Alzheimer’s disease. The Lancet (World Report).

[8] Mauricio R, Benn C, Davis J, Dawson G, Dawson LA, Evans A (2019). Tackling gaps in developing life-changing treatments for dementia. Alzheimer's Dementia (New York, N Y).

[9] Posner H, Curiel R, Edgar C, Hendrix S, Liu E, Loewenstein DA (2017). Outcomes assessment in clinical trials of Alzheimer's disease and its precursors: readying for short-term and long-term clinical trial needs. Innov Clin Neurosci.

[10] Snyder PJ, Kahle-Wrobleski K, Brannan S, Miller DS, Schindler RJ, DeSanti S (2014). Assessing cognition and function in Alzheimer's disease clinical trials: do we have the right tools?. Alzheimers Dement.

[11] Harvey PD, Cosentino S, Curiel R, Goldberg TE, Kaye J, Loewenstein D (2017). Performance-based and observational assessments in clinical trials across the Alzheimer's disease spectrum. Innov Clin Neurosci.

[12] Cummings J, Lee G, Ritter A, Sabbagh M, Zhong K (2020). Alzheimer's disease drug development pipeline: 2020. Alzheimer's Dement Transl Res Clin Interv.

[13] Hung A, Schneider M, Lopez MH, McClellan M (2020). Preclinical alzheimer disease drug development: early considerations based on phase 3 clinical trials. J Manag Care Spec Pharm.

[14] FDA UFaDA (2018) Early Alzheimer’s disease: developing drugs for treatment guidance for industry 10.1016/j.trci.2018.11.004 PMC6804505 31650002

[15] EMA (2018) EMA. Guideline on the clinical investigation of medicines for the treatment of Alzheimer’s disease. Committee for Medicinal Products for Human Use (CHMP), pp 1–36

[16] Tochel C, Smith M, Baldwin H, Gustavsson A, Ly A, Bexelius C (2019). What outcomes are important to patients with mild cognitive impairment or Alzheimer's disease, their caregivers, and health-care professionals? A systematic review. Alzheimers Dement (Amst).

[17] Harding AJE, Morbey H, Ahmed F, Opdebeeck C, Lasrado R, Williamson PR (2019). What is important to people living with dementia?: the ‘long-list’ of outcome items in the development of a core outcome set for use in the evaluation of non-pharmacological community-based health and social care interventions. BMC Geriatr.

[18] Reilly ST, Harding AJE, Morbey H, Ahmed F, Williamson PR, Swarbrick C (2020). What is important to people with dementia living at home? A set of core outcome items for use in the evaluation of non-pharmacological community-based health and social care interventions. Age Ageing.

[19] Watson J, Saunders S, Muniz Terrera G, Ritchie C, Evans A, Luz S (2019). What matters to people with memory problems, healthy volunteers and health and social care professionals in the context of developing treatment to prevent Alzheimer's dementia? A qualitative study. Health Expect.

[20] Saunders S, Muniz-Terrera G, Watson J, Clarke CL, Luz S, Evans AR (2018). Participant outcomes and preferences in Alzheimer's disease clinical trials: the electronic Person-Specific Outcome Measure (ePSOM) development program. Alzheimers Dement (N Y).

[21] Mlinac ME, Feng MC (2016). Assessment of Activities of Daily Living, Self-Care, and Independence. Arch Clin Neuropsychol.

[22] Pennington J, Socher R, Manning C (2014) Glove: global vectors for word representation. In: Proceedings of the 2014 conference on empirical methods in natural language processing (EMNLP), pp 1532–1543

[23] Sharp ES, Gatz M (2011). Relationship between education and dementia: an updated systematic review. Alzheimer Dis Assoc Disord.

[24] Bosco A, Schneider J, Coleston-Shields DM, Jawahar K, Higgs P, Orrell M (2019). Agency in dementia care: systematic review and meta-ethnography. Int Psychogeriatr.

[25] Moore JW (2016). What is the sense of agency and why does it matter?. Front Psychol.

[26] Hughes CP, Berg L, Danziger WL, Coben LA, Martin RL (1982). A new clinical scale for the staging of dementia. Br J Psychiatry.

[27] Grill JD, Karlawish J (2017). Study partners should be required in preclinical Alzheimer's disease trials. Alzheimer's Res Ther.

[28] Nuño MM, Gillen DL, Grill JD, Alzheimer’s Disease Cooperative Study (2019). Study partner types and prediction of cognitive performance: implications to preclinical Alzheimer’s trials. Alzheimers Res Ther.

[29] Sheehan B (2012). Assessment scales in dementia. Ther Adv Neurol Disord.

[30] Dassel KB, Schmitt FA (2008). The impact of caregiver executive skills on reports of patient functioning. Gerontologist.

[31] Mougias AA, Christidi F, Kontogianni E, Skaltsounaki E, Politis A, Politis A (2018). Patient- and caregiver-related factors associated with caregiver assessed global deterioration scale scoring in demented patients. Curr Gerontol Geriatr Res.

[32] Sperling RA, Aisen PS, Beckett LA, Bennett DA, Craft S, Fagan AM (2011). Toward defining the preclinical stages of Alzheimer's disease: recommendations from the National Institute on Aging-Alzheimer's Association workgroups on diagnostic guidelines for Alzheimer's disease. Alzheimers Dement.

