# Supplementary material for: Impact of clinical symptoms and diagnosis: the electronic Person-Specific Outcome Measure (ePSOM) development programme
Source: J Patient Rep Outcomes. 2022 Apr 5;6:33. doi: 10.1186/s41687-022-00433-2 (PMC8982721; doi:10.1186/s41687-022-00433-2)
Supplement: Supplementary file 1 — Additional file 1: Appendix 1. Full list of most Frequent themes (Diagnosis group). [file 41687_2022_433_MOESM1_ESM.pdf]

Full list of most Frequent themes: Diagnosis group

	Theme	Diagnosis	%	No Diagnosis	%	chi	pval
1	Driving	102	5.16	520	3.89	6.85	0.01
2	Socialising	65	3.29	395	2.95	0.56	0.46
3	Reading	64	3.24	501	3.75	1.11	0.29
4	Walking	62	3.14	419	3.13	0.00	0.95
5	Follow a storyline	60	3.04	384	2.87	0.11	0.74
6	Friendships	59	2.99	480	3.59	1.68	0.19
7	Family connection	51	2.58	384	2.87	0.43	0.51
8	Conversation and chat	48	2.43	265	1.98	1.51	0.22
9	Gardening	43	2.18	275	2.06	0.07	0.79
10	Cooking	35	1.77	215	1.61	0.19	0.66
11	Mix family connections friendships	34	1.72	262	1.96	0.40	0.53
12	Use technology	30	1.52	305	2.28	4.34	0.04
13	Remembering	27	1.37	81	0.61	13.19	0.01
14	Remember past	26	1.32	106	0.79	4.93	0.03
15	Recognise people	26	1.32	132	0.99	1.52	0.22
16	Shopping	26	1.32	143	1.07	0.75	0.39
17	Feel wanted and needed	26	1.32	142	1.06	0.80	0.37
18	Understand current affairs	23	1.16	106	0.79	2.42	0.12
19	Communicate effectively	21	1.06	142	1.06	0.01	0.91
20	Give advice	20	1.01	116	0.87	0.26	0.61
21	Personal attributes and social skills	20	1.01	131	0.98	0.00	0.99
22	Maintain independence	19	0.96	155	1.16	0.44	0.51
23	Analyse and solve problems	19	0.96	116	0.87	0.08	0.77
24	Hobbies	19	0.96	85	0.64	2.25	0.13
25	Grandchildren	18	0.91	138	1.03	0.15	0.70
26	Dining	18	0.91	136	1.02	0.10	0.75
27	Confidence	18	0.91	66	0.49	4.77	0.03
28	Meaningful conversations	18	0.91	173	1.29	1.75	0.19
29	Singing	18	0.91	64	0.48	5.27	0.02
30	Music	17	0.86	145	1.08	0.63	0.43
31	Help others	16	0.81	98	0.73	0.05	0.82
32	Express opinions	16	0.81	34	0.25	14.69	0.01
33	Make decisions	16	0.81	142	1.06	0.84	0.36
34	Exercise	16	0.81	106	0.79	0.00	0.95
35	Manage finances	16	0.81	214	1.60	6.77	0.01
36	Mixtheatrecinema	15	0.76	92	0.69	0.04	0.83
37	Golf	15	0.76	60	0.45	2.80	0.09
38	Volunteering	15	0.76	157	1.17	2.32	0.13

39	Walk dogs	15	0.76	66	0.49	1.83	0.18
40	Take part in activities	14	0.71	88	0.66	0.01	0.91
41	Cognitive games	14	0.71	254	1.90	13.55	0.01
42	Rational thinking	14	0.71	79	0.59	0.22	0.64
43	Planning and organising skills	14	0.71	98	0.73	0.00	0.98
44	Caring responsibilities	13	0.66	47	0.35	3.40	0.07
45	Feel valued and self-worth	13	0.66	49	0.37	2.95	0.09
46	Ability to learn new skills	13	0.66	60	0.45	1.18	0.28
47	Cycling	12	0.61	80	0.60	0.01	0.91
48	Going on holidays	11	0.56	80	0.60	0.00	0.95
49	Leisure travel	11	0.56	82	0.61	0.02	0.88
50	Maintain dignity	10	0.51	41	0.31	1.51	0.22
51	Working	10	0.51	43	0.32	1.21	0.27
52	Writing	10	0.51	76	0.57	0.03	0.85
53	Plan future	10	0.51	75	0.56	0.02	0.89
54	Personal hygiene	10	0.51	87	0.65	0.37	0.55
55	Mental agility	9	0.46	63	0.47	0.01	0.94
56	Staying active	9	0.46	46	0.34	0.33	0.57
57	Good listener	9	0.46	77	0.58	0.26	0.61
58	Household chores	9	0.46	59	0.44	0.01	0.93
59	Live at home	9	0.46	85	0.64	0.65	0.42
60	Analytical skills	9	0.46	78	0.58	0.30	0.58
61	Pets	9	0.46	49	0.37	0.16	0.69
62	Support family	9	0.46	83	0.62	0.54	0.46
63	Sense of humour	8	0.40	68	0.51	0.19	0.66
64	Remember names	8	0.40	29	0.22	1.81	0.18
65	Follow a conversation	8	0.40	25	0.19	2.86	0.09
66	Swimming	8	0.40	76	0.57	0.57	0.45
67	Travelling	8	0.40	140	1.05	6.78	0.01
68	Sports	8	0.40	76	0.57	0.57	0.45
69	Mixboard games and cards	8	0.40	51	0.38	0.00	0.97
70	Gym	8	0.40	52	0.39	0.01	0.93
71	Debate politics	7	0.35	52	0.39	0.00	0.97
72	Organise home	7	0.35	15	0.11	5.46	0.02
73	Use toilet	7	0.35	35	0.26	0.25	0.61
74	Watch tv	7	0.35	77	0.58	1.17	0.28
75	Mountain sports	6	0.30	28	0.21	0.33	0.57
76	Creative activities	6	0.30	35	0.26	0.01	0.92
77	Play musical instruments	6	0.30	65	0.49	0.88	0.35
78	Contribute to conversation	6	0.30	28	0.21	0.33	0.57
79	Plan holidays	6	0.30	36	0.27	0.00	0.97
80	Manage appointments	6	0.30	14	0.10	3.82	0.05
81	Laughter and fun	6	0.30	20	0.15	1.59	0.21
82	Water sports	6	0.30	18	0.13	2.16	0.14
83	Group activities	6	0.30	16	0.12	2.89	0.09
84	Remember books	5	0.25	26	0.19	0.07	0.78

85	Dressing style	5	0.25	25	0.19	0.12	0.73
86	Sport watching	5	0.25	10	0.07	3.93	0.05
87	Mixgalleriesmuseums	5	0.25	22	0.16	0.35	0.56
88	Academic activities	5	0.25	29	0.22	0.00	0.95
89	Remember recent events	5	0.25	13	0.10	2.36	0.12
90	Follow tv and news	5	0.25	34	0.25	0.05	0.82
91	Needlework	5	0.25	82	0.61	3.35	0.07
92	Religious participation	5	0.25	90	0.67	4.28	0.04
93	Artwork	5	0.25	45	0.34	0.16	0.69
94	Mixcookingbaking	4	0.20	26	0.19	0.04	0.84
95	Remember peoples name and face	4	0.20	8	0.06	2.84	0.09
96	Follow instructions	4	0.20	10	0.07	1.84	0.18
97	Remember important dates	4	0.20	41	0.31	0.33	0.56
98	Make people laugh	4	0.20	25	0.19	0.02	0.90
99	Remember where put things	4	0.20	39	0.29	0.22	0.64
100	Contribute to family	4	0.20	39	0.29	0.22	0.64
101	Yoga	4	0.20	34	0.25	0.04	0.85
102	Concentrate and understand books	4	0.20	36	0.27	0.09	0.76
103	Coffee with friends	4	0.20	28	0.21	0.04	0.84
104	Gaming	3	0.15	17	0.13	0.00	0.96
105	Remember day to day things	3	0.15	18	0.13	0.02	0.89
106	Remember conversations	3	0.15	11	0.08	0.31	0.58
107	Spouse	3	0.15	38	0.28	0.69	0.41
108	Craftwork	3	0.15	31	0.23	0.20	0.65
109	Understand tv programmes	3	0.15	33	0.25	0.32	0.57
110	Mixcinematheatre	3	0.15	9	0.07	0.68	0.41
111	Capacity to understand text	3	0.15	62	0.46	3.27	0.07
112	Read music	3	0.15	14	0.10	0.05	0.82
113	Same person	3	0.15	15	0.11	0.02	0.90
114	Role in the community	3	0.15	53	0.40	2.20	0.14
115	Look good	3	0.15	31	0.23	0.20	0.65
116	Remember love	3	0.15	7	0.05	1.31	0.25
117	Mixconcertsfestivals	3	0.15	29	0.22	0.11	0.74
118	Diy	3	0.15	6	0.04	1.78	0.18
119	Woman's role in family	3	0.15	6	0.04	1.78	0.18
120	Dancing	3	0.15	27	0.20	0.04	0.84
121	Empathy	3	0.15	36	0.27	0.53	0.47
122	Assess complex issues	3	0.15	25	0.19	0.00	0.95
123	Prioritise and analyse tasks	2	0.10	22	0.16	0.13	0.72
124	Racket sports	2	0.10	19	0.14	0.02	0.89
125	Dancing	2	0.10	10	0.07	0.00	0.97
126	Retain a good memory	2	0.10	13	0.10	0.11	0.74
127	Plan meals	2	0.10	9	0.07	0.01	0.94
128	Traveling	2	0.10	24	0.18	0.25	0.62
129	Mixgardeningother	2	0.10	22	0.16	0.13	0.72
130	Exercise classes	2	0.10	15	0.11	0.05	0.82

131	Take care of things	2	0.10	27	0.20	0.47	0.49
132	Running	2	0.10	35	0.26	1.24	0.27
133	Situational awareness	2	0.10	9	0.07	0.01	0.94
134	Fishing	2	0.10	5	0.04	0.46	0.50
135	Use public transport	2	0.10	17	0.13	0.00	0.97
136	Share memories	2	0.10	17	0.13	0.00	0.97
137	Remember routes	2	0.10	18	0.13	0.00	0.96
138	Pilates	1	0.05	5	0.04	0.11	0.74
139	Not be a burden	1	0.05	9	0.07	0.04	0.84
140	Photography	1	0.05	8	0.06	0.12	0.73
141	Mental arithmetic	1	0.05	2	0.01	0.04	0.84
142	New friends	1	0.05	10	0.07	0.01	0.94
143	Control budgets	1	0.05	10	0.07	0.01	0.94
144	Contribute to society	1	0.05	6	0.04	0.21	0.65
145	Compassionate	1	0.05	0	0.00	0.00	0.00
146	Maintain contact	1	0.05	11	0.08	0.00	0.97
147	Listen to radio	1	0.05	16	0.12	0.25	0.62
148	Pub	1	0.05	20	0.15	0.62	0.43
149	Remembering words	1	0.05	11	0.08	0.00	0.97
150	Wit and humour	1	0.05	12	0.09	0.02	0.89
151	Intelligence	1	0.05	41	0.31	3.25	0.07
152	Run household	1	0.05	13	0.10	0.06	0.81
153	Plan family life	1	0.05	35	0.26	2.44	0.12
154	Kind and caring	1	0.05	22	0.16	0.83	0.36
155	Mixconcertstheatrecinemamuseums	0	0.00	21	0.16	0.00	0.00
156	Retain maths ability	0	0.00	5	0.04	0.00	0.00
157	Motherhood	0	0.00	5	0.04	0.00	0.00
158	Academic activites	0	0.00	23	0.17	0.00	0.00
159	Mixridinghorseridingbike	0	0.00	6	0.04	0.00	0.00
160	Good company	0	0.00	3	0.02	0.00	0.00
161	Mix love other	0	0.00	3	0.02	0.00	0.00
162	Hearing	0	0.00	3	0.02	0.00	0.00
163	Discuss literature and science	0	0.00	25	0.19	0.00	0.00
164	Play games	0	0.00	2	0.01	0.00	0.00
165	Camping	0	0.00	2	0.01	0.00	0.00
166	Winter sports	0	0.00	6	0.04	0.00	0.00
167	Sex life	0	0.00	7	0.05	0.00	0.00
168	Remain positive	0	0.00	6	0.04	0.00	0.00
169	Plan leisure time	0	0.00	44	0.33	0.00	0.00
170	Mixyogapilates	0	0.00	7	0.05	0.00	0.00
171	Manage diary	0	0.00	7	0.05	0.00	0.00
172	Follow a recipe	0	0.00	8	0.06	0.00	0.00
173	Make tea	0	0.00	8	0.06	0.00	0.00
174	In control of life	0	0.00	8	0.06	0.00	0.00
175	Getting dressed	0	0.00	16	0.12	0.00	0.00
176	Bowls	0	0.00	15	0.11	0.00	0.00

177	Happy and loved	0	0.00	13	0.10	0.00	0.00
178	Tai chi	0	0.00	9	0.07	0.00	0.00
179	Follow needlework pattern	0	0.00	12	0.09	0.00	0.00
180	Speak foreign languages	0	0.00	10	0.07	0.00	0.00
181	Baking	0	0.00	11	0.08	0.00	0.00
182	Entertain	0	0.00	11	0.08	0.00	0.00
