# Supplementary material for: Impact of clinical symptoms and diagnosis: the electronic Person-Specific Outcome Measure (ePSOM) development programme
Source: J Patient Rep Outcomes. 2022 Apr 5;6:33. doi: 10.1186/s41687-022-00433-2 (PMC8982721; doi:10.1186/s41687-022-00433-2)
Supplement: Supplementary file 2 — Additional file 2: Appendix 2. Full list of most Important themes (Diagnosis group). [file 41687_2022_433_MOESM2_ESM.pdf]

Full list of most Important themes: Diagnosis group

	Theme	Diagnosis	%	No Diagnosis	%	chi	pval
1	Driving	60	8.00	260	5.56	6.53	0.01
2	Family connection	30	4.00	187	4.00	0.01	0.92
3	Conversation and chat	26	3.47	92	1.97	6.16	0.01
4	Reading	24	3.20	192	4.10	1.15	0.28
5	Friendships	24	3.20	182	3.89	0.66	0.42
6	Walking	24	3.20	151	3.23	0.01	0.94
7	Socialising	22	2.93	187	4.00	1.69	0.19
8	Mix family connections friendships	17	2.27	104	2.22	0.00	0.95
9	Remembering	15	2.00	45	0.96	5.46	0.02
10	Maintain independence	15	2.00	109	2.33	0.18	0.67
11	Communicate effectively	13	1.73	77	1.65	0.00	0.98
12	Feel wanted and needed	12	1.60	68	1.45	0.02	0.88
13	Use technology	12	1.60	104	2.22	0.92	0.34
14	Make decisions	10	1.33	53	1.13	0.09	0.77
15	Gardening	10	1.33	73	1.56	0.10	0.76
16	Cooking	10	1.33	65	1.39	0.00	0.96
17	Remember past	9	1.20	28	0.60	2.63	0.11
18	Dining	9	1.20	23	0.49	4.40	0.04
19	Exercise	9	1.20	47	1.00	0.09	0.77
20	Singing	8	1.07	25	0.53	2.22	0.14
21	Maintain dignity	8	1.07	24	0.51	2.51	0.11
22	Hobbies	8	1.07	36	0.77	0.39	0.53
23	Follow a storyline	8	1.07	87	1.86	1.92	0.17
24	Meaningful conversations	8	1.07	65	1.39	0.29	0.59
25	Recognise people	7	0.93	64	1.37	0.64	0.42
26	Grandchildren	7	0.93	53	1.13	0.09	0.77
27	Shopping	7	0.93	38	0.81	0.02	0.90
28	Walk dogs	6	0.80	30	0.64	0.07	0.80
29	Volunteering	6	0.80	54	1.15	0.45	0.50
30	Cognitive games	6	0.80	45	0.96	0.05	0.82
31	Golf	6	0.80	17	0.36	1.98	0.16
32	Going on holidays	6	0.80	19	0.41	1.41	0.23
33	Mixboard games and cards	5	0.67	18	0.38	0.64	0.42
34	Pets	5	0.67	21	0.45	0.27	0.60
35	Staying active	5	0.67	26	0.56	0.01	0.91
36	Live at home	5	0.67	52	1.11	0.84	0.36
37	Analyse and solve problems	5	0.67	33	0.71	0.01	0.91
38	Give advice	5	0.67	38	0.81	0.04	0.85

39	Understand current affairs	4	0.53	22	0.47	0.00	0.96
40	Travelling	4	0.53	35	0.75	0.17	0.68
41	Writing	4	0.53	21	0.45	0.00	0.98
42	Working	4	0.53	13	0.28	0.66	0.42
43	Mixtheatrecinema	4	0.53	9	0.19	1.88	0.17
44	Organise home	4	0.53	1	0.02	13.27	0.01
45	Personal attributes and social skills	4	0.53	34	0.73	0.12	0.72
46	Cycling	4	0.53	24	0.51	0.04	0.84
47	Sports	4	0.53	25	0.53	0.07	0.79
48	Caring responsibilities	4	0.53	25	0.53	0.07	0.79
49	Music	4	0.53	50	1.07	1.38	0.24
50	Express opinions	4	0.53	9	0.19	1.88	0.17
51	Personal hygiene	4	0.53	55	1.18	1.92	0.17
52	Use toilet	4	0.53	21	0.45	0.00	0.98
53	Confidence	3	0.40	18	0.38	0.06	0.80
54	Gym	3	0.40	16	0.34	0.01	0.93
55	Follow a conversation	3	0.40	7	0.15	1.05	0.30
56	Ability to learn new skills	3	0.40	14	0.30	0.01	0.91
57	Plan holidays	3	0.40	4	0.09	2.82	0.09
58	Follow tv and news	3	0.40	14	0.30	0.01	0.91
59	Remember names	3	0.40	5	0.11	2.05	0.15
60	Planning and organising skills	3	0.40	18	0.38	0.06	0.80
61	Mountain sports	3	0.40	10	0.21	0.32	0.57
62	Look good	3	0.40	29	0.62	0.22	0.64
63	Household chores	3	0.40	19	0.41	0.08	0.78
64	Manage finances	3	0.40	88	1.88	7.72	0.01
65	Support family	3	0.40	29	0.62	0.22	0.64
66	Mental agility	2	0.27	18	0.38	0.03	0.86
67	Gaming	2	0.27	5	0.11	0.34	0.56
68	Concentrate and understand books	2	0.27	8	0.17	0.01	0.91
69	Make people laugh	2	0.27	8	0.17	0.01	0.91
70	Retain a good memory	2	0.27	7	0.15	0.06	0.80
71	Assess complex issues	2	0.27	6	0.13	0.16	0.69
72	Mixgardeningother	2	0.27	6	0.13	0.16	0.69
73	Religious participation	2	0.27	34	0.73	1.44	0.23
74	Contribute to family	2	0.27	11	0.24	0.06	0.81
75	Prioritise and analyse tasks	2	0.27	3	0.06	1.10	0.29
76	Remember routes	2	0.27	3	0.06	1.10	0.29
77	Same person	2	0.27	2	0.04	1.89	0.17
78	Remember conversations	2	0.27	2	0.04	1.89	0.17
79	Remember love	2	0.27	2	0.04	1.89	0.17
80	Sport watching	2	0.27	1	0.02	3.30	0.07
81	Help others	2	0.27	26	0.56	0.56	0.45
82	Sense of humour	2	0.27	33	0.71	1.32	0.25
83	Diy	2	0.27	0	0.00	0.00	0.00
84	Take part in activities	2	0.27	19	0.41	0.06	0.80

85	Traveling	2	0.27	13	0.28	0.10	0.75
86	Remember where put things	2	0.27	17	0.36	0.01	0.93
87	Empathy	2	0.27	17	0.36	0.01	0.93
88	Leisure travel	2	0.27	20	0.43	0.11	0.74
89	Good listener	2	0.27	19	0.41	0.06	0.80
90	Situational awareness	1	0.13	4	0.09	0.06	0.80
91	Group activities	1	0.13	5	0.11	0.15	0.70
92	Feel valued and self-worth	1	0.13	18	0.38	0.56	0.45
93	Role in the community	1	0.13	15	0.32	0.27	0.61
94	Mixcookingbaking	1	0.13	5	0.11	0.15	0.70
95	Not be a burden	1	0.13	5	0.11	0.15	0.70
96	Remember recent events	1	0.13	4	0.09	0.06	0.80
97	Fishing	1	0.13	4	0.09	0.06	0.80
98	Running	1	0.13	11	0.24	0.02	0.90
99	Remember peoples name and face	1	0.13	3	0.06	0.01	0.94
100	Creative activities	1	0.13	16	0.34	0.36	0.55
101	Remember important dates	1	0.13	6	0.13	0.26	0.61
102	Needlework	1	0.13	16	0.34	0.36	0.55
103	Capacity to understand text	1	0.13	17	0.36	0.46	0.50
104	Mixcinematheatre	1	0.13	2	0.04	0.02	0.89
105	Racket sports	1	0.13	2	0.04	0.02	0.89
106	Water sports	1	0.13	2	0.04	0.02	0.89
107	Plan meals	1	0.13	1	0.02	0.21	0.65
108	Manage appointments	1	0.13	1	0.02	0.21	0.65
109	Spouse	1	0.13	19	0.41	0.67	0.41
110	Swimming	1	0.13	15	0.32	0.27	0.61
111	Take care of things	1	0.13	11	0.24	0.02	0.90
112	Kind and caring	1	0.13	8	0.17	0.06	0.80
113	Laughter and fun	1	0.13	9	0.19	0.01	0.91
114	Play musical instruments	1	0.13	24	0.51	1.29	0.26
115	Analytical skills	1	0.13	21	0.45	0.91	0.34
116	Dancing	1	0.13	8	0.17	0.06	0.80
117	Plan future	1	0.13	15	0.32	0.27	0.61
118	Understand tv programmes	1	0.13	7	0.15	0.16	0.69
119	Debate politics	1	0.13	11	0.24	0.02	0.90
120	Academic activities	1	0.13	8	0.17	0.06	0.80
121	Craftwork	1	0.13	12	0.26	0.06	0.81
122	Remember books	1	0.13	10	0.21	0.00	0.99
123	Contribute to conversation	1	0.13	7	0.15	0.16	0.69
124	Wit and humour	1	0.13	6	0.13	0.26	0.61
125	Yoga	1	0.13	8	0.17	0.06	0.80
126	Artwork	1	0.13	13	0.28	0.11	0.74
127	Coffee with friends	1	0.13	11	0.24	0.02	0.90
128	Rational thinking	0	0.00	21	0.45	0.00	0.00
129	Baking	0	0.00	2	0.04	0.00	0.00
130	New friends	0	0.00	2	0.04	0.00	0.00

131	Hearing	0	0.00	2	0.04	0.00	0.00
132	Follow needlework pattern	0	0.00	2	0.04	0.00	0.00
133	Retain maths ability	0	0.00	2	0.04	0.00	0.00
134	Exercise classes	0	0.00	6	0.13	0.00	0.00
135	Discuss literature and science	0	0.00	2	0.04	0.00	0.00
136	Mixridinghorseridingbike	0	0.00	1	0.02	0.00	0.00
137	Entertain	0	0.00	2	0.04	0.00	0.00
138	Remain positive	0	0.00	1	0.02	0.00	0.00
139	Photography	0	0.00	1	0.02	0.00	0.00
140	Follow a recipe	0	0.00	1	0.02	0.00	0.00
141	Mix love other	0	0.00	1	0.02	0.00	0.00
142	Tai chi	0	0.00	1	0.02	0.00	0.00
143	Listen to radio	0	0.00	1	0.02	0.00	0.00
144	Control budgets	0	0.00	1	0.02	0.00	0.00
145	Good company	0	0.00	1	0.02	0.00	0.00
146	Camping	0	0.00	1	0.02	0.00	0.00
147	Happy and loved	0	0.00	2	0.04	0.00	0.00
148	Use public transport	0	0.00	8	0.17	0.00	0.00
149	In control of life	0	0.00	2	0.04	0.00	0.00
150	Academic activites	0	0.00	8	0.17	0.00	0.00
151	Pub	0	0.00	6	0.13	0.00	0.00
152	Maintain contact	0	0.00	6	0.13	0.00	0.00
153	Dressing style	0	0.00	6	0.13	0.00	0.00
154	Mixconcertstheatrecinemamuseums	0	0.00	5	0.11	0.00	0.00
155	Watch tv	0	0.00	15	0.32	0.00	0.00
156	Run household	0	0.00	5	0.11	0.00	0.00
157	Intelligence	0	0.00	5	0.11	0.00	0.00
158	Remember day to day things	0	0.00	7	0.15	0.00	0.00
159	Bowls	0	0.00	7	0.15	0.00	0.00
160	Make tea	0	0.00	5	0.11	0.00	0.00
161	Share memories	0	0.00	4	0.09	0.00	0.00
162	Getting dressed	0	0.00	8	0.17	0.00	0.00
163	Pilates	0	0.00	2	0.04	0.00	0.00
164	Sex life	0	0.00	4	0.09	0.00	0.00
165	Dancing	0	0.00	4	0.09	0.00	0.00
166	Plan leisure time	0	0.00	4	0.09	0.00	0.00
167	Mixgalleriesmuseums	0	0.00	3	0.06	0.00	0.00
168	Read music	0	0.00	3	0.06	0.00	0.00
169	Remembering words	0	0.00	3	0.06	0.00	0.00
170	Speak foreign languages	0	0.00	3	0.06	0.00	0.00
171	Motherhood	0	0.00	2	0.04	0.00	0.00
172	Plan family life	0	0.00	6	0.13	0.00	0.00
173	Mixconcertsfestivals	0	0.00	2	0.04	0.00	0.00
174	Woman's role in family	0	0.00	2	0.04	0.00	0.00
175	Mixyogapilates	0	0.00	4	0.09	0.00	0.00
